# Carboxymethyl Chitosan Microgels for Sustained Delivery of Vancomycin and Long-Lasting Antibacterial Effects

**DOI:** 10.3390/gels9090708

**Published:** 2023-09-01

**Authors:** Mehtap Sahiner, Aynur S. Yilmaz, Ramesh S. Ayyala, Nurettin Sahiner

**Affiliations:** 1 Department of Bioengineering, Faculty of Engineering, Canakkale, Onsekiz Mart University Terzioglu Campus, Canakkale 17100, Turkey; sahinerm78@gmail.com; 2 Department of Chemical, Biological and Materials Engineering, University of South Florida, Tampa, FL 33620, USA; sanemyilmazz99@gmail.com; 3 Department of Chemistry, Faculty of Sciences, and Nanoscience and Technology Research and Application Center (NANORAC), Canakkale Onsekiz Mart University Terzioglu Campus, Canakkale 17100, Turkey; 4 Department of Ophthalmology, Morsani College of Medicine, University of South Florida Eye Institute, 12901 Bruce B Down Blvd., MDC 21, Tampa, FL 33612, USA; rsayyala@gmail.com

**Keywords:** carboxymethyl chitosan, microgel, sustained drug delivery, antibiotic, Fe (II) chelating activity, vancomycin, prolonged antibacterial effect

## Abstract

Carboxymethyl chitosan (CMCh) is a unique polysaccharide with functional groups that can develop positive and negative charges due to the abundant numbers of amine and carboxylic acid groups. CMCh is widely used in different areas due to its excellent biocompatibility, biodegradability, water solubility, and chelating ability. CMCh microgels were synthesized in a microemulsion environment using divinyl sulfone (DVS) as a crosslinking agent. CMCh microgel with tailored size and zeta potential values were obtained in a single stem by crosslinking CMCh in a water-in-oil environment. The spherical microgel structure is confirmed by SEM analysis. The sizes of CMCh microgels varied from one micrometer to tens of micrometers. The isoelectric point of CMCh microgels was determined as pH 4.4. Biocompatibility of CMCh microgels was verified on L929 fibroblasts with 96.5 ± 1.5% cell viability at 1 mg/mL concentration. The drug-carrying abilities of CMCh microgels were evaluated by loading Vancomycin (Van) antibiotic as a model drug. Furthermore, the antibacterial activity efficiency of Van-loaded CMCh microgels (Van@CMCh) was investigated. The MIC values of the released drug from Van@CMCh microgels were found to be 68.6 and 7.95 µg/mL against *E. coli* and S. *aureus*, respectively, at 24 h contact time. Disk diffusion tests confirmed that Van@CMCh microgels, especially for Gram-positive (*S. aureus*) bacteria, revealed long-lasting inhibitory effects on bacteria growth up to 72 h.

## 1. Introduction

Carboxymethyl chitosan (CMCh) is a chitosan (Ch) derivative composed of glucosamine units [1]. Ch is a biocompatible, biodegradable, non-toxic, antimicrobial, accessible natural polymer and has been used in biomedical applications due to its gene-carrying ability, bacteria growth-inhibitory effects, anti-oxidant and anti-inflammatory activities [1,2]. However, the low solubility of Ch in an aqueous environment restricts these biological properties [3]. Certain functional groups, such as amino and hydroxyl groups added onto Ch chains, allow Ch to be chemically modified so that the resultant structure possesses improved water solubility, allowing Ch derivatives multiple functions to be explored for new applications. Carboxymethylation is one of the methods for hydrophilic modification, and CMCh is proven to have numerous better biological properties and unique abilities, including wound healing capability, mucoadhesive, gelling, metal-ion chelating, moisture retention ability as well as injectability, bioimaging, gene and enzyme delivery aptitudes making CMCh as one of the most preferred materials in tissue engineering applications with its additional advantages such as degradability [1,4,5]. Depending on the carboxymethylation reaction, O-Carboxymethyl chitosan, N, O-Carboxymethyl chitosan, and N, N-Carboxymethyl chitosan with different biological activities can be obtained [6,7]. CMCh-based gels were found to be biocompatible with mesenchymal stem cells, osteoblasts, and fibroblasts [8,9,10]. Due to the presence of a higher number of chelating groups, supramolecular assemblies of CMCh, i.e., CMCh-based hydrogels, can increase the moisture retention time, improve water solubility, biodegradability, and enhance antibacterial activity [11,12]. Moreover, CMCh, as a drug carrier, is able to exhibit slower drug release and better adsorption ability compared to CH, which directly affects its bioavailability [13].

Novel gel-based drug carriers derived from natural polymers have been proposed for the treatment of a wide variety of diseases, including neurological disorders, diabetes, infections, psoriasis, glaucoma, and cancer [14,15,16,17], and the majority of them showed promising results. Considering the higher cost of manufacturing processes of synthetic drug molecules or drug delivery systems, the ease of production and modification of nature-based materials such as CH and CMCh are quite advantageous, especially in the biomedical, cosmetic, and food industries [1,18,19]. In literature, CMCh-based scaffolds, nanoparticles, and hydrogels showed controllable pore size, higher swelling ability, degradability, cell adhesion, and proliferation capabilities, e.g., Rao et al. reported that poly(vinyl alcohol) and CMC-containing wound dressing material and showed that these materials have high cell viability on connective tissue cells [20]. Another recently published study indicated that CMCh had to promote wound healing in burn wounds, such as wound contraction [18]. Local delivery of chemotherapeutics and their sustained release for up to 5 days was achieved using CMCh-based hydrogels [21]. In addition, CMCh showed pH-responsive swelling ability and high biocompatibility in normal cells. Recently, the controlled release of drugs from carriers via different factors such as pH, temperature, electrical field, and partition coefficient has become a highly preferred method [14,16,22]. Natural polymers such as CMCh can integrate and release drugs at a higher rate with their unique bio-beneficial properties with good swelling ability, entrapment, and degradation properties, maintaining the relevant drug concentration in the blood for an extended period [23,24]. CMCh-based polymeric structures were reported to be used for the delivery of a variety of antimicrobial agents such as ciprofloxacin [22], fluconazole [25], clindamycin [4], gentamycin sulfate [5], voriconazole [26]. To date, biocompatible Ch hydrogels crosslinked with different agents such as glutaraldehyde, formaldehyde, and oxalic acid for tissue engineering are reported [27,28]. Despite these gel-based polymeric structures that possess porous and interconnected structure, elasticity, flexibility, and moisturize-retention ability, the low mechanical strength, poor stability, and insolubility of hydrogels at certain pH conditions significantly restrict their use [27]. Likewise, the standardization of CS-based hydrogels, as well as their insufficient clinical utility, persist as substantial limitations [29]. On the other hand, nano- and microgels of chemically crosslinked Ch such as Ch:methacrylates, CMCh:sodium tripolyphosphate complex, CMCh:alginate complex, and CH:gelatin composites were proven to overcome most of these limitations and exhibit improved properties for drug delivery purposes including injectability [30,31,32].

Vancomycin (Van) is a first-generation glycopeptide antibiotic especially prescribed for hospital-acquired infections [33]. Van is suggested in many diseases such as colitis, skin and soft tissue infections, and endocarditis with FDA approval. The mechanism of action of Van is to stop the cell wall production of bacteria by binding to peptidoglycan precursors and inhibiting the protein–biosynthesis cycle. Hence, in addition to the main effects on Gram-positive bacteria, Van also has broad-spectrum antimicrobial effects. Van is administered parenterally or orally, but due to its hydrophilic nature, Van is released fast and has limited membrane permeability. Low systemic absorption (oral bioavailability of less than 10%) [34] and rapid clearance (elimination half-life is 5–11 h range) [35] of this drug are the important obstacles, and it was reported that Van should be used with a loading dose to ensure effective blood levels [36]. However, severe diarrhea, hearing loss, angioedema, neutropenia, nephrotoxicity, ototoxicity, and Van flushing syndrome (VFS, with quite varying incidences, were reported as non-negligible adverse effects of this antibiotic. These adverse effects strongly concern patients with diabetes, renal impairment, and hypersensitivity [35,37].

Several methods for Van loading into mesoporous materials or gel-based polymeric structures, including physical mixing and vacuum-assisted loading, were reported [38] to increase the bioavailability of Van. These reports confirmed that Van release from Van-loaded particles could last more than 5 days [38]. Poly(ethylene glycol) conjugated Van showed high drug loading content and stability [33]. Since Van is known as a critical situation medicine, it is important to provide optimum delivery conditions for this antibiotic. Rapid Van release, along with a prolonged local drug concentration for killing the bacteria in the initial stage, is preferred to obtain a better therapeutic response as well as to prevent continuous bacterial infections [39]. Van is a large antibiotic having approximately 1500 Da of molecular weight and a big molecular size (3.2 × 2.2 nm) [40,41]. Therefore, the potential Van carriers should possess appropriate characteristics, e.g., size, pore, functional groups, etc., to encapsulate this drug as well as have controllable swelling or degradability capabilities. To date, Van@hydroxy propyl methyl cellulose microparticles [42], Van-encapsulated poly(epsilon-caprolactone) microparticles [43], Van@poly (lactic-co-glycolic acid)(PLGA) microspheres [44] with sustained release of Van up to 6.5, 7 and 28 days, respectively, are reported. In a recent study conducted by Yu et al., Van@PLGA microspheres with pH-responsive characteristics were proposed [45]. It was proven that Van-loaded microspheres were able to inhibit bacteria growth of *S. aureus* even on the 50th day of the drug release.

Here, we report CMCh microgels as a biocompatible drug delivery system designed for carrying active agents such as antibiotics, antifungals, antivirals, antineoplastic agents, and so on. Microgels of CMCh were synthesized in a microemulsion system. Functional and morphological characterization of CMCh microgels was elucidated by FTIR spectroscopy and scanning electron microscope (SEM) images. The hydrolytic degradation of the prepared microgels was determined by pH-dependent weight loss studies. In addition, the biocompatibility of CMCh microgels was assessed on connective tissue cells in vitro. Moreover, Van (Van HCl) as a model drug was loaded into CMCh microgels, and its release characteristics were studied because of the rapid elimination and low bioavailability nature of this antibiotic. The main aim of Van loading into CMCh microgels was to obtain a sustained drug release profile lasting more than 24 h. Furthermore, the antibacterial activity of CMCh microgels and its drug-loaded form were tested against both Gram-negative *Escherichia coli* and Gram-positive *Staphylococcus aureus*. Further, the prolonged drug release was planned to be confirmed by antimicrobial activity studies in vitro.

## 2. Results and Discussion

**Figure 1:**
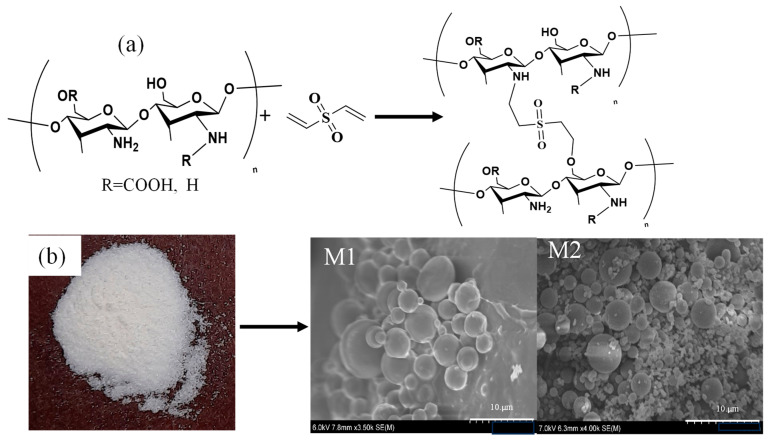
(**a**) Schematic presentation of CMCh microgels preparation and (**b**) photograph of powder CMCh molecules and the SEM images of CMCh microgels synthesized in M1 (0.2 M AOT/isooctane medium) and M2 (0.1 M lecithin/cyclohexane) medium, respectively.

DVS forms ether bonds by the reaction of the primary hydroxyl groups of glucose units, e.g., in Hyaluronic acid) [46] or by reaction of amine groups, e.g., in the preparation of polyethyleneimine particles [47] under alkaline conditions. The reaction of DVS as a crosslinker in crosslinking is reaction is rapid, resulting in gel formation within minutes [48]. A schematic diagram of the synthesis of CMCh microgels with DVS is illustrated in Figure 1. It is apparent that spherical-shaped CMCh microgels with smooth surface morphology are successfully formed in two different reaction mediums. The SEM images of microgels synthesized in two different microenvironments are given in Figure 1. The dimensions of the microgels were determined using the ImageJ program. The microgels synthesized in 0.2 M AOT/isooctane medium (M1) were 5.8 ± 4.8 µm in size, while the microgels synthesized in 0.1 M lecithin/cyclohexane medium (M2) were found to be in 1.2 ± 0.4 µm. The yield was less than 25% in the M1 medium, whereas the yield was 89.4 + 3.3% for the microgels prepared in M2. Therefore, the studied for the progress of this research were continued using CMCh particles prepared in M2 medium.

The SEM images of Van@CMCh microgels are given in Figure S1. Although the CMCh microgels have a smooth spherical shape, the Van@CMCh microgels have reduced roundness and smoothness. The presence of drug within the network of CMCh upon drying can cause this kind of alteration of the smooth surface features.

**Figure 2:**
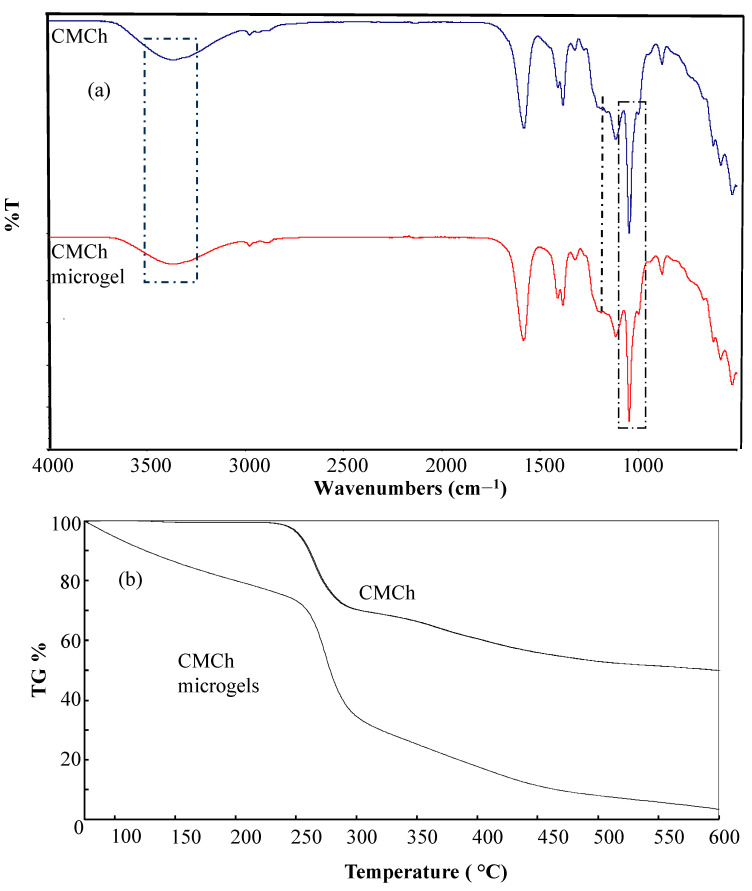
(**a**) FT-IR spectrum of CMCh and CMCh microgels and (**b**) and their thermal degradation (TG %) curves.

From the FT-IR spectra of CMCh and CMCh microgels shown in Figure 2a, the wide-stretching peak in the 3200–3550 cm^−1^ band is noticeable for both CMCh, i.e., for CMCh polymer and its microgel. It has been reported that DVS shows an FT-IR peak at 1312 cm^−1^ (S=O asymmetric stretching vibrations) and at 1131 cm^−1^ for S=O symmetric stretching vibrations [47]. Since the S=O (asymmetric stretching vibrations) peak of DVS seen in 1310 overlaps with the O-H peak of CHCh, and the S=O (symmetric stretching vibrations) peak in 1100 coincides with the ether peak, -CH_2_-O- of CMCh, no new peak is visualized upon microgel formation by DVS crosslinking of CMCh. It is also evident that some of the stretching frequencies of CMCh and crosslinked CMCh microgel peaks overlapped, such as S=O stretching, C-O stretching, and O-H vibrations. On the other hand, the peak intensity belonging to R-O-R upon crosslinking of -OH groups of CHCh molecules with DVS at around 1100 cm^−1^ is increased.

The thermal gravimetric analysis provides a quantitative measurement of weight change as the temperature is increased. Figure 2b shows a thermal gravimetric analysis of CMCh and CMCh microgels. In the thermal degradation graph of CMCh, about 1.2 wt% at 239 °C and about 29 wt% at 291 °C were degraded, and heating up to 600 °C, 49.8 wt% of CMCh remained. The CMCh microgels, on the other hand, retained 73.5 wt% of their initial weight at 249.1 °C. At 303.6 °C, 33.5% of its weight remained, and at about 600 °C, 3.4% of the weight of the CMCh microgels remained.

**Figure 3:**
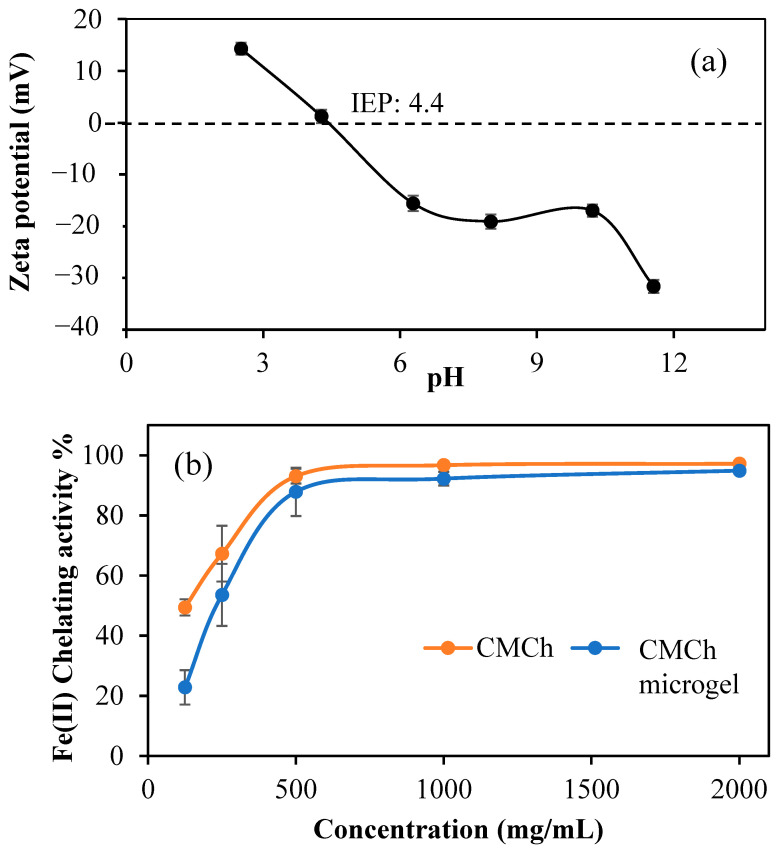
(**a**) Zeta potential measurements of CMCh microgels at various pHs in 0.01 M KNO_3_ solutions and (**b**) Fe(II) chelating activity % of CMCh and CMCh microgels.

In a 1 mM KNO_3_ solution, in varying pH (2.5–11.5) solutions, the zeta potential values of CMCh microgels’ were investigated. The pH was found to be 10.22, and the zeta potential was −16.9 ± 1.2 mV for 20 mg/mL microgel in 1 mM KNO_3_. The zeta potentials against changing pH are given in Figure 3a. Based on these data, the isoelectric point (IEP) was calculated as pH 4.4. When the IEP is greater than 7, the surface is called a basic surface. When the IEP is less than 7, the surface is called an acidic surface. In this study, CMCh microgels IEP is less than 7. This indicates that the surface of the CMCh microgels has an acidic character. From this point of view, it can be deduced that the acidic characters due to -OH and -COOH groups on the surface are more than the basic characters such as -NH_2_ groups.

Fe(II) chelating activity of CMCh and CMCh microgels was investigated in the 125–2000 mg/mL concentration range. As seen in Figure 3b, Fe(II) chelating capability of both CMCh and CMCh microgels increases depending on the concentration of microgels. CMCh and CMCh microgel chelate Fe(II) ions at very high rates. At 250 mg/mL concentration, CMCh chelates 67.3 ± 9.3% of Fe(II) ion, while CMCh microgel chelates only 53.6 ± 10.3% of it. This can be understandable because of the crosslinked structure of CMCh microgels; some of the carboxylic acid amine groups from CMCh chains are not readily available for chelation with Fe(II) due to the microgel network.

**Figure 4:**
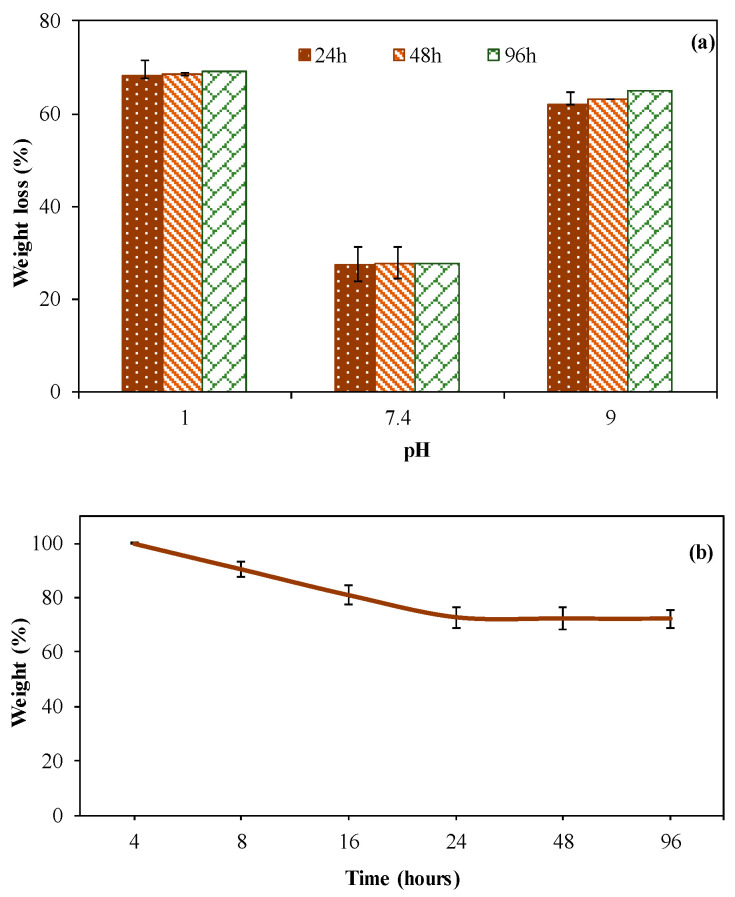
(**a**) Weight loss (%) of CMCh microgels at different pH conditions at 24 h, 48 h and 72 h incubation times and (**b**) gravimetric weight loss (%) of CMCh microgels with time at pH 7.4, 37 °C.

The hydrolytic degradation of CMCh microgels was investigated by gravimetric analysis at pH 1, 7.4, and 9 as simulation environments for the stomach [49], physiological conditions [49], and duodenum [50] conditions, respectively. The incubation times of 24 h, 48 h, and 72 h were chosen, and their results are illustrated in Figure 4a as the weight loss (%) of the microgels.

As seen in Figure 4a, the weight loss (%) of CMCh microgels was determined as 68.1% ± 3.7%, 27.3% ± 4.0%, 62.1% ± 2.3% at pH 1, 7.4, and 9, respectively, in 24 h. These results indicated that CMCh microgels are highly degradable under acidic (pH 1) and basic (pH 9) conditions. Further, it was found that CMCh microgels are highly stable under physiological conditions, pH 7.4. The hydrolytic degradation of CMCh microgels was also analyzed for longer incubation times, 48 h and 72 h. According to the degradation results, 68.4% ± 2.4%, 27.7% ± 4.1%, 63.2% ± 0.5% weight loss (%) at 48 h incubation, and 69.1% ± 0.5%, 27.8% ± 3.0%, 64.7% ± 2.9% were measured for 96 h incubation times at pH 1, 7.4, and 9, respectively. As seen, more than 50% of the microgels were degraded within the first 24 h at pH 1 and pH 9, indicating that hydrolytic degradation of CMCh microgels was in line with the desired degradation profile for certain applications. Considering the regional pH changes observed in the human body due to reasons such as inadequate blood perfusion, tumor formation, hypoxia, and inflammation [51,52], controllable drug release from CMCh microgels could be obtained by endogenous stimuli. For example, the tumor microenvironment is more acidic (pH 5.6 to 6.8) compared to physiological pH [53]. Therefore, it can be said that pH-dependent drug release for targeting certain body parts could be achieved by drug-loaded CMCh microgels with suitable drug loads. Furthermore, the degradation rate of the microgels did not change significantly between 24 h and 72 h, revealing that CMCh microgels can remain stable for up to 3 days after degradation at a certain rate. In addition, the hydrolytic degradation of CMCh microgels was performed at pH 7.4 and 37 °C at various times, as shown in Figure 4b. As seen at 4 h, no degradation of CMCh microgels was observed. It was observed that, at 8 h, 16 h, 24 h, 48 h, and 96 h incubation times, 9.57%, 19.1%, 27.3%, 27.78%, and 27.81% of the weight loss were observed, respectively, for the prepared CMCh microgels. Furthermore, by reducing the crosslinker ratio, microgels with higher degradability can be obtained [54]. The degradation profiles of 10% and 25% crosslinked CMCh microgels, which can be degraded at a higher rate, will be investigated in our future studies for different applications.

Fibroblasts are differentiated into many cell types, including adipogenic, chondrogenic, and osteogenic cells [55]. L929 fibroblasts are adhesive cells derived from Mouse C3/An connective tissue. L929 fibroblasts, with their reproducible biological responses and susceptibility to toxic effects, are considered correct cells for biocompatibility studies [56]. Therefore, CMCh and CMCh microgel samples up to 1000 µg/mL concentrations were used in biocompatibility studies on L929 fibroblasts at 24 h incubation time.

**Figure 5:**
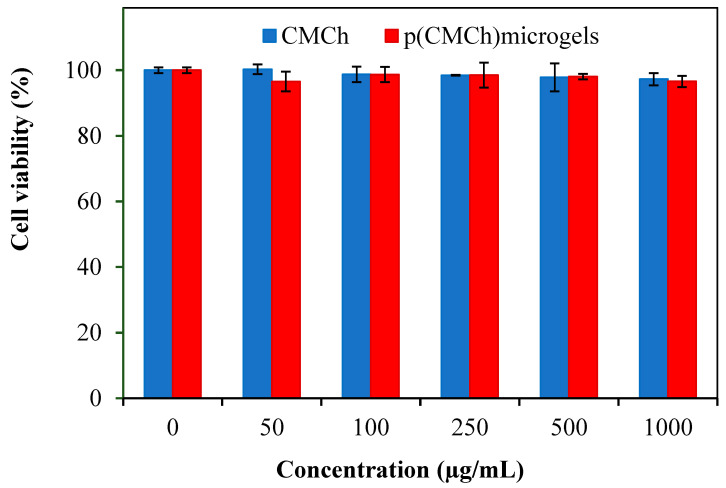
In vitro biocompatibility studies of CMCh molecules and microgels at 24 h incubation time.

As illustrated in Figure 5, the cell viability (%) values of the natural CMCh polymer and CMCh microgels were found to be 97.2 ± 1.8% and 96.6 ± 1.7%, respectively, at the highest concentration, 1 mg/mL. According to the biocompatibility test data and the Anova statistical test results, there is no statistical difference between each (50–1000 ug/mL) concentration of CMCh and the control. For CMCh microgels, it was not statistically different from the control at all concentrations. Therefore, prepared microgels, with their excellent cell compatibility, allow them to be designed and safely used for biological, biomedical, and pharmaceutical applications.

**Figure 6:**
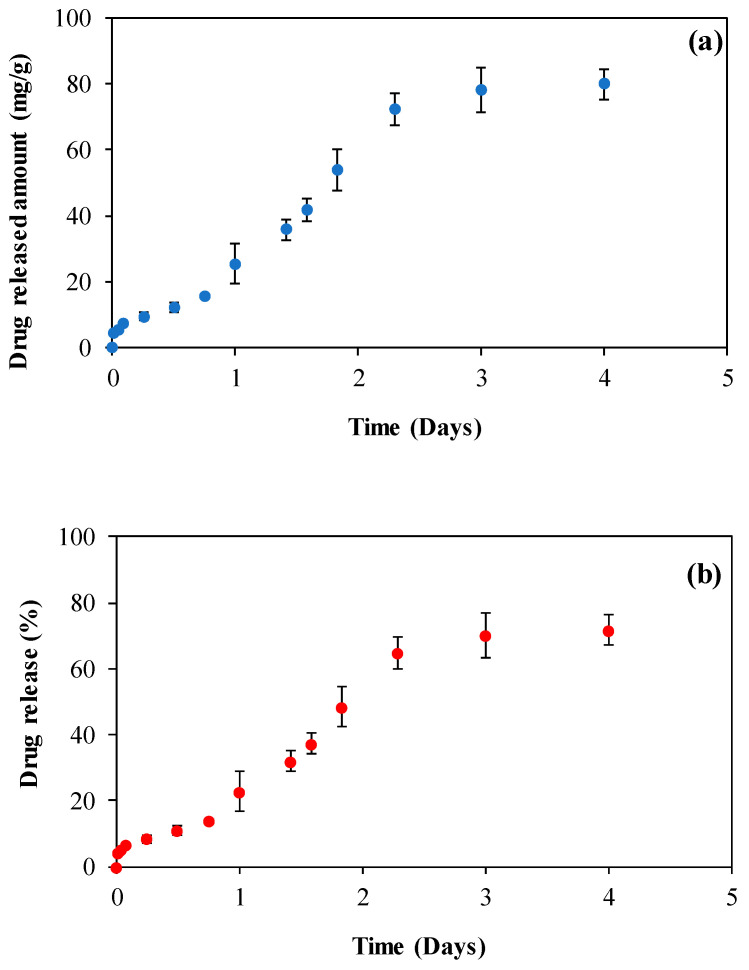
(**a**) Vancomycin (Van) release profile (mg/g) with time and (**b**) Van release (%) within 4 days.

Van is loaded into CMCh microgels for a short amount of time, 2 h, by adsorption method. Van loading amount of CMCh microgels was found to be 111.42 ± 7.08 mg/g. As previously stated in the previous sections, Van is a large active pharmaceutical compound antibiotic. Here, a high amount of drug loading for Van was reported. Van-loaded microgels (Van@CMCh) underwent SEM analysis to further examine the microgels in their drug-incorporated forms, and the corresponding images are given in Figure S1. The drug-loading process was carried out in an aqueous solution, and the loaded particles were washed with ethanol solution. In Figure S1, it is seen that the spherical shape of the prepared materials did not change after Van loading. It assumed that Van drug molecules (seen as smaller particles) are homogenously distributed into CMCh microgels. Van release studies from Van@CMCh microgels were performed at pH 7.4 and 37 °C in vitro, and the corresponding graphs are illustrated in Figure 6.

As seen in Figure 6, within the first hour, the sixth hour, and twelfth hour of the drug release, 5.55 ± 0.1, 9.35 ± 1.1, and 12.14 ± 1.3 mg/g Van were released from Van@CMCh microgels which equal to 4.98%, 8.38% and 10.89% of the loaded Van, respectively. It can be said that even up to 30 h, 4.6 ± 0.1 mg/g (4.13% of the loaded antibiotic) was released, which indicates the rapid release of the antibiotic. Van release from Van@CMCh microgels was linear up to 50 h, whereas from 50 h to 96 h, the drug released was found at a considerably slower rate. Overall, 79.99 mg/g Van was released from Van@CMCh microgels, which shows that 71.8% of the loaded drug was released within 4 days, which also fits the desired amount. The calibration curve of Van in PBS solution at 280 nm is given in Figure S2. Moreover, different kinetic models such as zero order, first order, Higuchi, and Korsmeyer–Peppas models were applied to the drug release graphs to examine the Van release from CMCh microgels. The calculated values for each model were summarized in Table 1, and the corresponding release (%) vs time (day) plots for related models are given in Figure S3.

Correlation coefficients (R^2^) shown in Table 1 indicate that the Korsmeyer–Peppas kinetic model fitted Van release the best with a higher R^2^ value, 0.9763. As seen, the Higuchi model was the one fitted with the lowest R^2^ value of 0.8257. The R^2^ values of the zero-order and first-order models for Van release are calculated as 0.9652 and 0.9264, respectively. The Korsmeyer–Peppas kinetic model is defined as a linear and non-linear regression model in which the n values determine the drug release mechanism [57]. Korsmeyer–Peppas model is reported to be applicable for the release of various drugs from the drug-loaded polymeric structures [58]. The n values for Van release from Van@CMCh microgels were calculated as 1.245, which exceeds the value of 0.89 and reveals that the release model fitted the super case II transport [57,58].

Antibacterial potencies of CMCh, CMCh microgels, Van@CMCh, and Van were tested against two common bacteria strains, *E. coli* and *S. aureus*, and the corresponding results were summarized in Table 2.

As shown in Table 2, CMCh and CMCh microgels did not show any antibacterial effect on the studied microorganisms up to 2.5 mg/mL concentrations. On the other hand, on the well plate, transparent wells (an indicator of no visible growth) containing Van@CMCh microgels were detected, and MIC and MBC values were determined accordingly. MIC values of Van released from Van@CMCh microgels were found to be 64 µg/mL and 8 µg/mL for *E. coli* and *S. aureus*, respectively. Moreover, MBC values of Van released from Van@CMCh microgels were found to be 8 µg/mL. Micro-titer assay results are also illustrated in Figure S4 as the reduction in the colony-forming units (expressed logarithmically). For *E. coli*, no concentration was detected that kills 99.9% of the microorganism within 24 h. These results are correlated with the literature because Van is mainly effective on Gram-positive bacteria and has mild antibacterial effects on Gram-negative bacteria [34]. Further, bare Van was tested as a control. MIC and MBC values of bare-Van were found to be 15 µg/mL and 250 µg/mL for *E. coli* and 0.2 µg/mL and 0.2 µg/mL for *S. aureus*, respectively.

**Figure 7:**
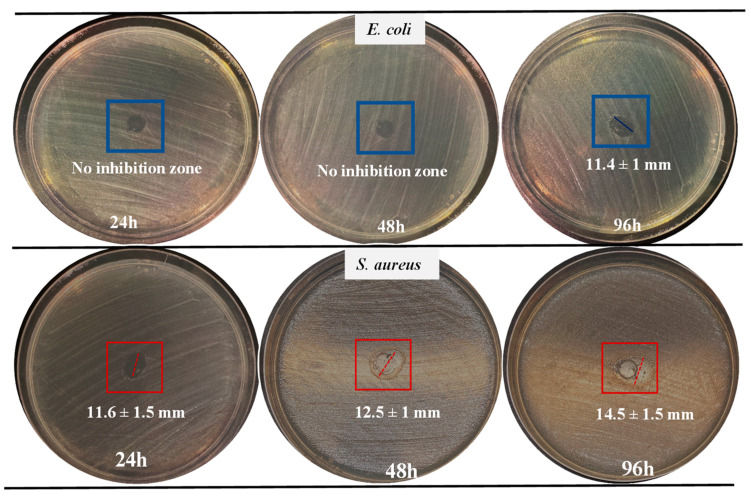
Photographs of inhibition zone diameters of Van@CMCh microgels (20 µL of 5 mg/mL) at 24 h, 48 h, and 96 h incubation times.

Van@CMCh microgel suspension at 5 mg/mL concentration was freshly prepared, and 20 µL of the suspension was placed onto disks and incubated for 24 h, 48 h, and 72 h at 35 °C. The inhibition zone diameters and photographs of the disk diffusion assay are given in Table 3 and Figure 7, respectively.

The inhibition zone for *E. coli* was detected only at 96 h as 11.4 ± 1 mm. Inhibition zone diameters for *S. aureus* were measured as 11.6 ± 1.5, 12.5 ± 1, and 14.5 ± 1.5 mm at 24 h, 48 h, and 72 h incubation times, respectively. It can be noticed that for *S. aureus*, especially at 48 h and 96 h, a relatively less symmetrical zone was detected due to the retention of the particle suspension on the disc, as can be seen in Figure 7. However, since the endpoint where bacteria could grow was seen, the zones were measured with average values. Disk diffusion test results are strongly correlated with the in vitro drug release studies of Van@CMCh microgels. The inhibition zones for *S. aureus* from 24 h to 72 h expanded significantly, revealing that the drug release continued, and the antibacterial effect lasted at least for 72 h. One of the areas where extended drug release is most needed is the administration of antimicrobial agents. Some of the antibiotics and antifungals currently used have become insufficiently effective due to unnecessary use, inadequate or ill-timed dosing, enzymatic inactivation of drugs, changes in drug targets, and excretion of drugs by active transport proteins in microorganisms [59]. Antimicrobial resistance, especially seen in bacterial species such as *Escherichia coli*, *Staphylococcus aureus*, *Klebsiella pneumoniae*, and *Streptococcus pneumonia*, reveals difficult cases to treat, such as nosocomial infections [60]. Under selective antibiotic pressure, drug-susceptible bacteria are destroyed or stop growing, while naturally resistant bacteria can survive [61]. Therefore, it is crucial that therapeutic agents are given at appropriate intervals and exhibit improved absorption and distribution profiles [62]. The extended release of certain antibiotics, such as Van, could improve their in vivo half-life and that bioavailability. In healthy adults, the terminal half-life of Van is reported as 4–6 h [34].

In the current study, Van release from CMCh microgels lasted for a significantly longer time (4 days), revealing that the prepared microgels were found quite successful as antibiotic carriers. Furthermore, wide-spectrum antibiotics, as well as anticancer, antifungal, antiviral, anti-inflammatory, antihistaminic drugs, and so on, can be loaded into CMCh microgels. Considering their degradability profile, controlled drug release for pH or temperature-sensitive drugs can also be readily achievable.

## 3. Conclusions

In this study, the single-step preparation of CMCh microgels via a microemulsion method by crosslinking with DVS was reported. The microsphere formation was confirmed via SEM and FT-IR spectroscopy analyses. The prepared CMCh microgels are spherical and 1.2 ± 0.4 μm size range. Despite the ease of their production, the possible residue from the organic solvent and chemicals can be identified as a limitation. Hence, the microemulsion polymerization technique requires a proper microgel/particle washing process to remove the surfactant from the environment, or the surfactant-free synthesis method needs to be considered for in vivo applications. The hydrolytic stability of CMCh microgels was confirmed that the microgel has a pH dependent on degradation profiles and can degrade up to about 70% at pH 1 and 9 while degrading about 30% at pH 7.4 up to 96 h contact times. The cell cytotoxicity results of CMCh microgels performed on L929 fibroblasts indicated that prepared CMCh microgels did not induce any significant toxicity even at 1 mg/mL concentration with cell viability values more than 95%. Moreover, the drug delivery efficiency of CMCh microgels was evaluated using Van, a large antibiotic with a rapid clearance profile and low bioavailability, as a model drug. Van-loaded CMCh microgels showed sustained drug release up to 96 h. Furthermore, the prolonged Van release ability of CMCh microgels was confirmed by antimicrobial activity studies on *E. coli* and *S. aureus* bacteria. The high drug loading capability of CMCh microgels (111.42 ± 7.08 mg/g) suggests that other large drug molecules or drugs with stability and solubility issues could be delivered utilizing CMCh microgels as highly biocompatible drug delivery vehicles. Further, the drug release amount and kinetic can be readily controlled by a suitable amount of crosslinker used during particle preparation. Moreover, the higher surface area of microgels provides many advantages over common bulk hydrogel formulations. Therefore, CMCh microgels, with their adjustable degradability, pore characteristics, and controllable drug loading and release properties, have many advantages for various drugs with limited activities in the treatment of different diseases.

## 4. Materials and Methods

### 4.1. Materials

Carboxymethyl chitosan (Santa Cruz Biotechnology, Fischer Scientific, Deacetylation degree 90%, Hampton, New Hampshire) as a starting material and divinyl sulfone (DVS, >96%, TCI) as a chemical crosslinker was used in CMCh microgel preparation. L-alpha-Lecithin, granular, from soybean oil (Across), sodium bis(2-ethylhexyl) sulfosuccinate (AOT, 96%, Sigma Aldrich, St. Louis, MO, USA) as a surfactant, and Cyclohexane (Certified ACS, Fisher Chemical™, Pittsburgh, PA, USA, 99+%), 2,2,4-trimethylpentane (isooctane, Sigma) as a solvent were used as received in CMCh microgels preparation Vancomycin hydrochloride (Van HCl, Alfa Aesar, Thermo Fisher Scientific, Molecular Biology Grade, Waltham, MA, USA) was purchased and used as a model antibiotic for drug delivery studies. For the iron (II) chelating assay, 3-(2-pyridyl)-5,6-diphenyl-1,2,4-triazine-4′,4′′-disulfonic acid sodium salt (ferrozine, ≥98%, from Santa Cruz Biotechnology, Dallas, TX, USA) and iron (II) sulfate heptahydrate (FeSO_4_∙7H_2_O, >99.5%, ACS reagent from Across Organics, Geel, Belgium) were used as received. For the cell viability tests, the L929 fibroblast cell line was obtained from the SAP Institute (Ankara, Turkey). Dulbecco’s Modified Eagle’s Medium (DMEM/F-12, 1:1) (L-Glutamine, 15 mM HEPES, 1.2 g/L NaHCO_3_) as the cell culture medium was purchased from Pan Biontech GmbH, Aidenbach, Germany. Fetal bovine serum (FBS), antibiotic solution (penicillin–streptomycin), and trypsin-EDTA (0.25%) were used as received (Pan Biontech GmbH, Aidenbach, Germany). Trypan Blue (0.5% solution) was acquired from Biological Industries, and thiazolyl blue tetrazolium bromide (MTT) was obtained from BioFroxx (Einhausen, Germany). Dimethyl sulfoxide (DMSO, 99.9%, Carlo-Erba, Val-de-Reuil, France) was used as received. Molecular porous membrane tubing was obtained from Spectrum Laboratories (MWCO: 12-14 kD, Fischer Scientific, San Jose, CA, USA). For antibacterial activity tests, Gram-negative bacteria *Escherichia coli* ATCC 8739 and Gram-positive bacteria *Staphylococcus aureus* ATCC 6538 were obtained from KWIK-STIK™ Microbiologics (St. Cloud, MN, USA). Nutrient agar and nutrient broth as growth medium were purchased from BD Difco ^TM^ (Becton, Dickinson and Company, Sparks, MD, USA) and used as received.

### 4.2. Synthesis and Characterization of CMCh Microgels

CMCh microgels were prepared by micro emission method in two different environments. Briefly, 0.05 g CMCh was dissolved in 1.5 mL 0.5 M NaOH solution. A total of 0.5 mL of this solution was placed in 0.1 M lecithin/cyclohexane medium. Then, 50 moles of crosslinking agent, DVS was put into the mixed solution. After an hour, the solution mixture was precipitated at 1000 rpm for 10 min. The CMCh microgels were washed 2 times with cyclohexane, 2 times with ethanol and 2 times with an ethanol: water (1:1) mixture. Finally, it was washed once with acetone.

Similarly, 0.5 mL of the CMCh solution was put into 0.2 M AOT/isooctane medium, and 50% DVS crosslinker was added and allowed to react for 1 h. The precipitation process was achieved by centrifuging twice at 1000 rpm with acetone.

Using the attenuated total reflection (ATR) technique, the spectra of CMCh and CMCh microgels were evaluated using Fourier transform infrared radiation (FT-IR, Nicolet iS10, Thermo, Boston, MA, USA). A thermogravimetric analyzer determined the percentage of CMCh microgels (exstar, SII TG/DTA6300, Seiko Ins. Corp, Tokyo, Japan). About 5 mg of CMCh sample was heated from 100 to 600 °C with a temperature increase of 10 °C/min under the influence of nitrogen gas flow of 200 mL/min for thermogravimetric analysis.

In 40 mL of 10 Mm KNO_3_ solution, 20 mg of CMCh microgels were suspended. Zeta potential measurements performed in 40 mL of 1 mM KNO_3_ solution to determine the surface charge of the microgels.

In accordance with the literature, chelating Fe (II) was performed [63]. For this purpose, 96 well plates were filled with 140 μL of CMCh and CMCh microgels. A 20 mL of 1 mM Fe(ll) aqueous solution was then added to the sample and measured at 562 nm with a Thermo Multiscan Go microplate reader. A second reading was performed after adding 40 μL of 2.5 mM ferrozine solution. The following formula was used to calculate Fe (II) chelating activity.

$$\mathrm{Iron}\;(\mathrm{II})\;\mathrm{chelating}\;\mathrm{activity}\%=\left[1-\frac{\Delta A_{562}^{sample}}{\Delta A_{562}^{control}}\right]\;\times \;100$$

### 4.3. Degradation Profile of CMCh Microgels

The stability and degradation profiles of CMCh microgels were investigated by hydrolytic degradation studies at 24 h, 48 h, and 72 h incubation times at different solution pH values, pH 1, 7.4, and 9. For this goal, CMCh microgels weighing 20 mg were placed in 20 mL of buffered pH solutions of pH 1, 7.4, and 9, in centrifuge tubes and kept up to three days. At certain times, at 24 h, 48 h, and 72 h incubation times, samples were taken and centrifuged at 10,000 rpm for 15 min to precipitate the non-degraded CMCh microgels. Then, the supernatant was gently decanted, and the precipitated particles were dried in an oven at 50 °C overnight. Weight loss (%) was calculated by the difference between the initial microgel weight and the microgel weights at 24 h, 48 h, and 72 h incubation periods. Moreover, hydrolytic weight loss kinetics of CMCh microgels were studied in detail at pH 7.4 and 37 °C at 4 h, 8 h, 16 h, 24 h, 48 h and 96 h incubation. Hydrolytic degradation studies were performed three times, and the mean values are given with the standard deviations.

### 4.4. In Vitro Cell Compatibility Studies of CMCh Microgels

The cell viability of CMCh microgels was determined on the L929 fibroblast cell line via colorimetric MTT assay following the literature [64]. CMCh stock solution and CMCh microgel suspensions were prepared by weighing 10 mg of each CMCh-based material and suspending them in 10 mL of DMEM solution. Initial concentrations of CMCh-based samples were prepared at 1 mg/mL and diluted in DMEM to prepare different concentrations of samples. The fibroblasts were cultured in a DMEM medium containing 10% FBS and 1% antibiotic for 4 days. For the cytotoxicity analysis, 100 μL of cell suspension containing 1 × 10^3^ cells/mL were seeded onto a 96-well plate and incubated at 37 °C in a 5% CO_2_/95% air atmosphere. After 24 h incubation, cells were checked and interacted with 100 μL of CMCh-based samples at 0.05–1 mg/mL concentrations. After 24 h of the incubation period, the old culture media containing samples was discarded, and the cells were washed with phosphate buffer saline (PBS) solution two times. Then, 0.1 mL of fresh prepared MTT solution at 0.5 mg/mL was placed onto each well and incubated at 37 °C for 3 h in a dark condition. After this period, formazan crystals produced by the active mitochondria in viable cells were dissolved using 0.2 mL of DMSO and slowly mixed. After 20 min, the absorbance values of the wells were measured at 570 nm by using a plate reader (Thermo Scientific, Multiskan Sky, Waltham, MA, USA). GraphPad Prism 9 software was used for the statistical analysis of cytotoxicity analysis. Statistical differences between the control groups and samples were measured using Dunnett’s multiple-comparison test and one-way ANOVA. A *p*-value < 0.05 was considered statistically significant.

### 4.5. Drug Delivery Abilities of CMCh Microgels

The drug-loading process of Vancomycin (Van) into CMCh microgels was completed by the soaking method (adsorption technique), as described in the literature [65]. Briefly, 0.1 g of CMCh microgels were immersed in 30 mL of 1 mg/mL Van aqueous solution and stirred at 5000 rpm for 2 h. The drugs can be loaded into polymeric particles for long periods, i.e., 12 h and 24 h [66], but the Van loading process was performed for a shorter amount of time (2 h) in order not to degrade 50% crosslinked microgels during the drug loading time. After Van loading, antibiotic-loaded microgels as Van@CMCh were precipitated in the same medium at 10,000 rpm for 5 min, then washed once with an ethanol solution to eliminate the drug molecules that adhered to the outer surface but did not penetrate the microgel structure. Finally, Van@CMCh microgels were dried at 50 °C oven overnight.

In vitro drug release studies of Van@CMCh, microgels proceeded at 37 °C and physiological pH condition (pH 7.4) to mimic the normal body temperature. First, Van@CMCh microgels of 50 mg were weighed and suspended in 1 mL of phosphate-buffered saline solution (PBS, sterilized) in dialysis tubing. Then, Van@CMCh containing dialysis membrane was placed in 20 mL of PBS solution in falcon tubes and kept in a shaking bath. Uv-Vis spectra were recorded at various times, i.e., each measurement was performed three times, and the results are given as the average values. The loaded and released amounts of antibiotic drug were calculated by using the calibration curve of Van at 280 nm in DI water and PBS solutions, respectively, via UV-Vis spectroscopy.

### 4.6. Antibacterial Activities of Van@CMCh Microgels

Bacteria growth inhibition and bactericidal effects of Van@CMCh were investigated against Gram-negative bacteria *E. coli* ATCC 8739 and gram-positive bacteria *S. aureus* ATCC 6538 by micro-titer dilution and inhibition zone assays as described in the literature [67,68]. CMCh, CMCh microgels, and Van@CMCh microgels weighing 50 mg were sterilized under UV irradiation at 355 nm and then suspended in 10 mL of PBS solution. Microgel-containing samples were sonicated for 30 s for homogenization and then immediately used.

#### 4.6.1. Broth Micro-Titer Dilution Assay

The bacterial suspensions were adjusted in nutrient broth (NB) to a McFarland standard of 0.5, which corresponds to 1.5 × 10^8^ CFU/mL [69]. Then, 0.1 mL of NB was added to each well of the 96-well plate. Then, 0.1 mL of CMCh-based samples were placed in the first well of each column on the plate and diluted with the existing media. Lastly, 10 µL of bacteria inoculum at 0.5 McFarland was added to each well and gently mixed. A vancomycin aqueous solution of 1 mg/mL was used as a control. Bacteria containing well-plates were incubated at 35 °C for 24 h. After this time, MIC and MBC values of CMCh-based materials were determined as the concentration that showed no visible growth in the wells and killed 99.9% of the microorganisms, respectively.

#### 4.6.2. Zone of Inhibition Method

Following the micro-titer assay, zone of inhibition experiments were performed for Van@CMCh microgels against both bacteria strains at 24 h, 48 h, and 72 h incubation periods. For this, 100 µL of *E. coli* and *S. aureus* inoculums at 0.5 McFarland were poured onto nutrient agar solid growth medium on petri dishes. Then, sterilized 10 mm × 10 mm spherical-shaped filter papers were placed on the petri dishes. Immediately, 20 µL of Van@CMCh microgel suspensions at 5 mg/mL were gently placed onto filter papers and incubated at 35 °C for three days. After the incubation, disks were taken, and the inhibition zones observed around the disks were measured.

## Figures and Tables

**Table 1:** The list of kinetic models and parameters calculated for the release of Van from Van@CMCh microgels.

Kinetic Model	Parameters	Van@CMCh Microgels
Zero order	k_0_	25.582
	R^2^	0.9652
First order	k_1_	0.327
	R^2^	0.9264
Higuchi	k_H_	30.667
	R^2^	0.8257
Korsmeyer–Peppas	k_KP_	22.437
	n	1.245
	R^2^	0.9763

**Table 2:** MIC and MBC values of CMCh-based materials.

Microorganisms	*E. coli*		*S. aureus*	
Sample	MIC (µg/mL)	MBC (µg/mL)	MIC (µg/mL)	MBC (µg/mL)
CMCh	-	-	-	-
CMCh microgels	-	-	-	-
Van@CMCh microgels	64	-	8	8
Vancomycin *	15	250	0.2	0.2

* Vancomycin aqueous solution was used as control.

**Table 3:** Inhibition zone diameters of Van@CMCh microgels (20 µL of 5 mg/mL).

Inhibition Zone Diameter (mm)	24 h	48 h	96 h
*E. coli*	-	-	11.5 ± 1
*S. aureus*	11.6 ± 1.5	12.5 ± 1	14.5 ± 1.5

## Data Availability

The data presented in this study are available on request from the corresponding author.

## Supplementary Materials

The following supporting information can be downloaded at: https://www.mdpi.com/article/10.3390/gels9090708/s1, Figure S1: SEM images of Van@p(CMCh) microgels at different magnifications; Figure S2: The Calibration curve of Vancomycin (Van) in phosphate-buffered saline solution at 280 nm; Figure S3: The Van release% from Van@CMCh microgels vs. time plots for related kinetic models; Figure S4: Colony forming units (CFU/mL) of (a) *E. coli* and (b) *S. aureus* strains versus the concentration of the released amount of Van from Van@CMCh microgels at 24 h incubation time.
