# Supplementary material for: Carboxymethyl Chitosan Microgels for Sustained Delivery of Vancomycin and Long-Lasting Antibacterial Effects
Source: Gels. 2023 Sep 1;9(9):708. doi: 10.3390/gels9090708 (PMC10529976; doi:10.3390/gels9090708)
Supplement: Supplementary file 1 [file gels-09-00708-s001.zip › gels-2574548-supplementary.pdf]

Supporting Information

Carboxymethyl chitosan microgels for sustained delivery of Vancomycin and long-lasting antibacterial effects

Mehtap Sahiner [1,2], Aynur S. Yilmaz [2,3], Ramesh S. Ayyala [4] and Nurettin Sahiner [2,3,4,*]

1 Department of Bioengineering, Faculty of Engineering, Canakkale, Onsekiz Mart University Terzioglu Campus, Canakkale, 17100, TURKEY; sahinerm78@gmail.com (M. Sahiner)
2 Department of Chemical, Biological and Materials Engineering, University of South Florida, Tampa, FL 33620, USA; sahinerm78@gmail.com (M. Sahiner); sanemyilmazz99@gmail.com (A.S. Yilmaz); sahiner71@gmail.com (N. Sahiner)
3 Department of Chemistry, Faculty of Sciences, and Nanoscience and Technology Research and Application Center (NANORAC), Canakkale Onsekiz Mart University Terzioglu Campus, Canakkale, 17100, TURKEY; sahinerm78@gmail.com (M. Sahiner); sanemyilmazz99@gmail.com (A.S. Yilmaz); sahiner71@gmail.com (N. Sahiner)
4 Department of Ophthalmology, Morsani College of Medicine, University of South Florida Eye Institute,12901 Bruce B Down Blvd, MDC 21, Tampa, FL 33612, USA; rsayyala@gmail.com (R.S. Ayyala); sahiner71@gmail.com (N. Sahiner)
* Correspondence: sahiner71@gmail.com; nsahiner@usf.edu

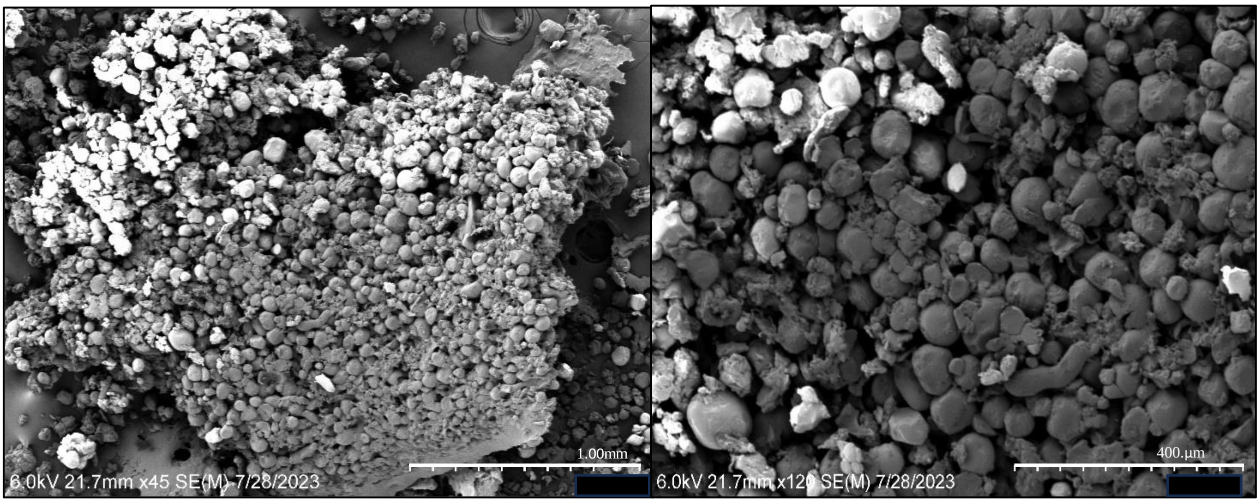

Figure S1. SEM images of Van@p(CMCh) microgels at different magnifications.

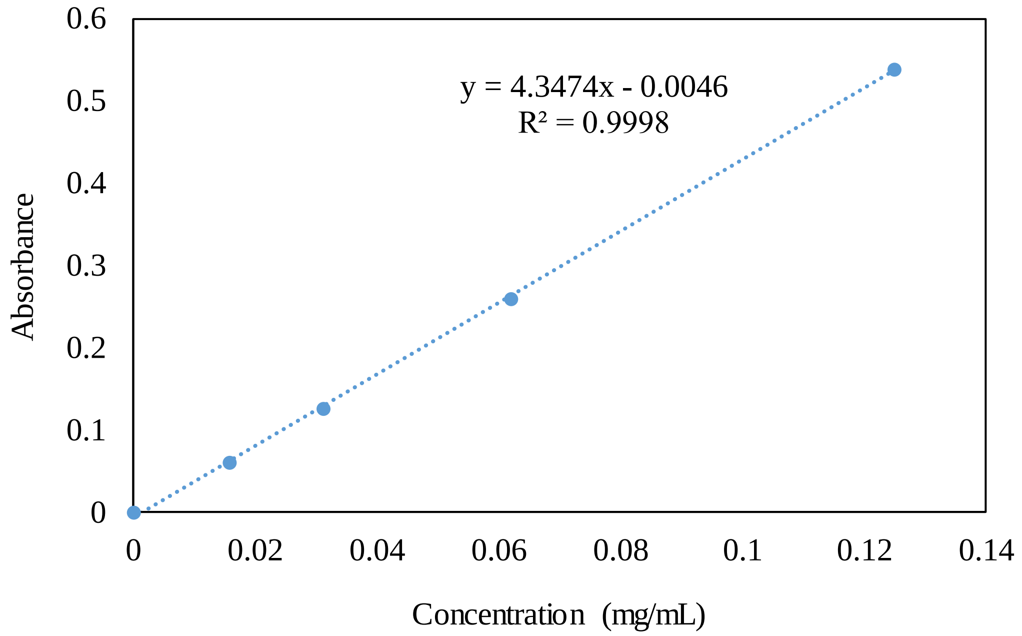

Figure S2. The Calibration curve of Vancomycin (Van) in phosphate-buffered saline solution at 280 nm.

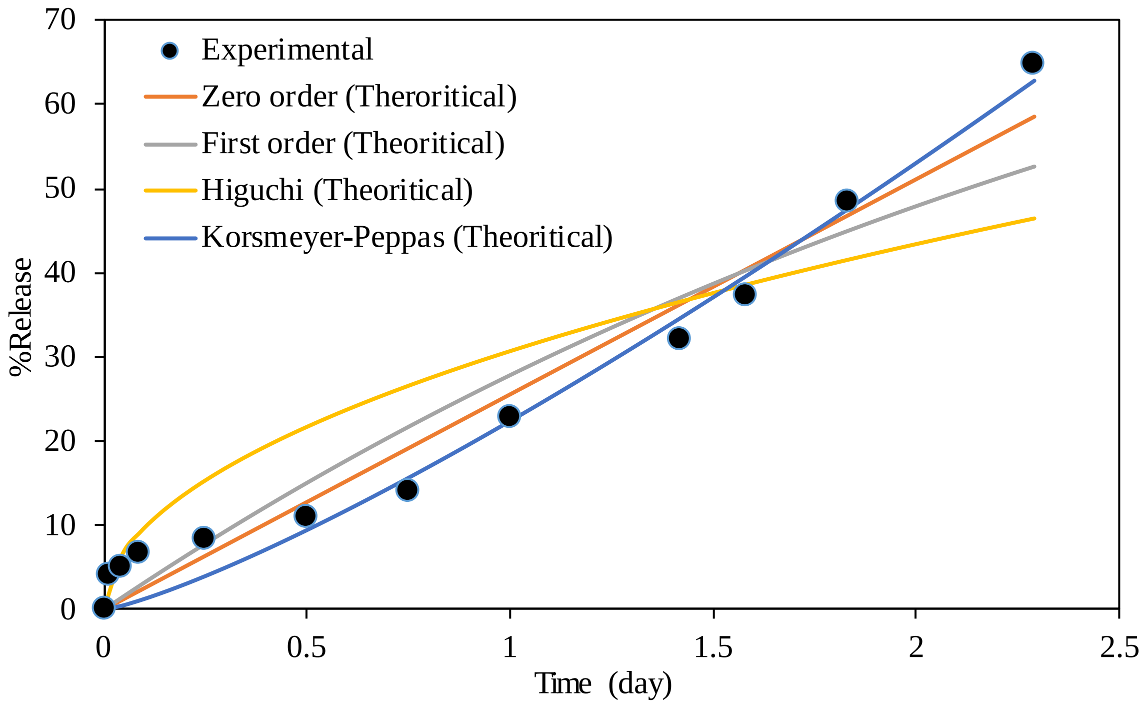

Figure S3. The Van release% from Van@CMCh microgels vs time plots for related kinetic models.

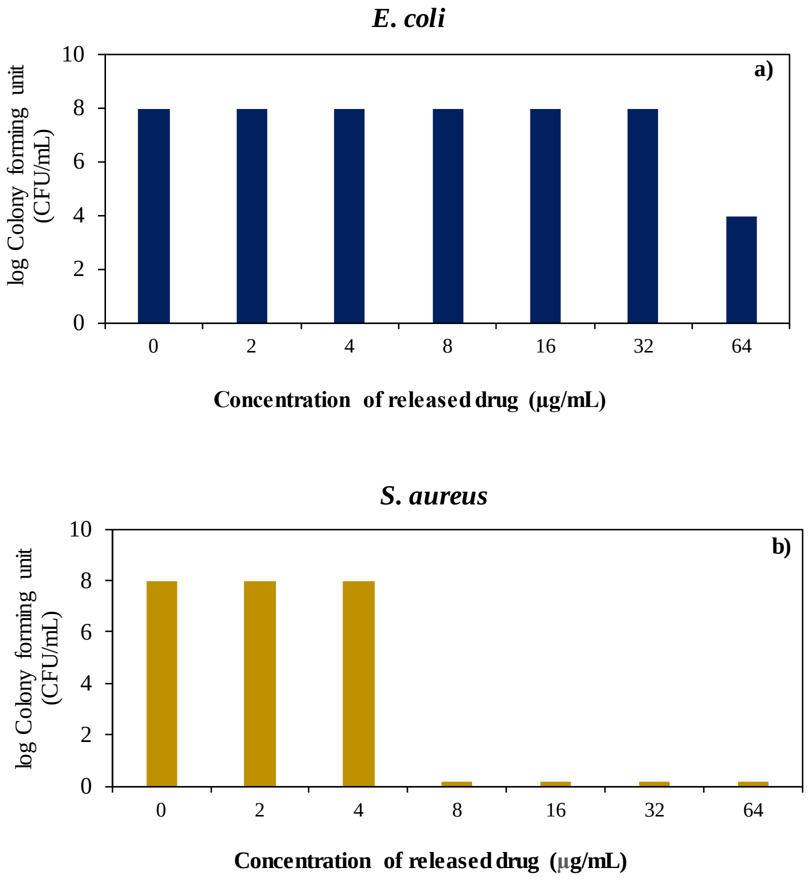

Figure S4. Colony forming units (CFU/mL) of (a) *E. coli* and (b) *S. aureus* strains versus the concentration of the released amount of Van from Van@CMCh microgels at 24h incubation time.
